# Clinical characteristics and outcome of lung cancer in patients with fibrosing interstitial lung disease

**DOI:** 10.1186/s12890-024-02946-6

**Published:** 2024-03-15

**Authors:** Soo Jin Han, Hyeon Hwa Kim, Dong-gon Hyun, Wonjun Ji, Chang-Min Choi, Jae Cheol Lee, Ho Cheol Kim

**Affiliations:** 1 Department of Pulmonary and Critical Care Medicine, Asan Medical Center, University of Ulsan College of Medicine, 88 Olympic-ro 43-gil, Songpa-gu, Seoul 05505 Republic of Korea; 2 Department of Oncology, Asan Medical Center, University of Ulsan College of Medicine, Seoul, Republic of Korea

**Keywords:** Idiopathic pulmonary fibrosis, Interstitial lung disease, KL-6, Lung cancer, Prognosis

## Abstract

**Background:**

Lung cancer (LC) is an important comorbidity of interstitial lung disease (ILD) and has a poor prognosis. The clinical characteristics and outcome of each ILD subtype in LC patients have not been sufficiently investigated. Therefore, this study aimed to evaluate the difference between idiopathic pulmonary fibrosis (IPF) and non-IPF ILD as well as prognostic factors in patients with ILD-LC.

**Methods:**

The medical records of 163 patients diagnosed with ILD-LC at Asan Medical Center from January 2018 to May 2023 were retrospectively reviewed. Baseline characteristics and clinical outcomes were compared between the IPF-LC and non-IPF ILD-LC groups, and prognostic factors were analyzed using the Cox proportional-hazard model.

**Results:**

The median follow-up period was 11 months after the cancer diagnosis. No statistically significant differences were observed in clinical characteristics and mortality rates (median survival: 26 vs. 20 months, *p* = 0.530) between the groups. The independent prognostic factors in patients with ILD-LC were higher level of Krebs von den Lungen-6 (≥ 1000 U/mL, hazard ratio [HR] 1.970, 95% confidence interval [CI] 1.026-3.783, *p* = 0.025) and advanced clinical stage of LC (compared with stage I, HR 3.876 for stage II, *p* = 0.025, HR 5.092 for stage III, *p* = 0.002, and HR 5.626 for stage IV, *p* = 0.002). In terms of treatment, surgery was the significant factor for survival (HR 0.235; 95% CI 0.106-0.520; *p* < 0.001).

**Conclusions:**

No survival difference was observed between IPF-LC and non-IPF ILD-LC patients. A higher level of Krebs von den Lungen-6 may act as a prognostic marker in ILD-LC patients.

**Supplementary Information:**

The online version contains supplementary material available at 10.1186/s12890-024-02946-6.

## Background

Interstitial lung diseases (ILDs) are a group of diffuse parenchymal lung disorders [1] that affect the pulmonary interstitial space [2]. An estimated number of more than 200 diseases have been reported to belong to ILD. Idiopathic pulmonary fibrosis (IPF), the most common type of fibrosing ILD, is a type of chronic and progressive disease of unknown cause [3] with a median survival of 3–5 years from the time of diagnosis [4]. Patients with IPF have several comorbidities, including pulmonary hypertension, emphysema, and lung cancer (LC) [5]. Among various comorbidities, LC has a reported prevalence of approximately 20% in patients with IPF [6], which is higher than in the general population [7]. Furthermore, among patients with IPF, the mean survival time was shorter (1.6-1.7 years) in those who had LC than in those who had no LC [8]. Recently, IPF and LC have been suggested to share common genetic and pathogenic mechanisms [9]. 

Although IPF is the most common type of ILD, it only accounts for 17-37% of all ILD diagnoses [10]. Recently, LC has been reported to be an important comorbidity in patients with ILD other than IPF [11, 12]. Furthermore, several previous studies have reported that the prevalence of LC is higher in non-IPF ILD patients than in the general population [13, 14]. However, the clinical characteristics of LC patients with ILD according to ILD subtypes have not been clearly elucidated. Therefore, this study aimed to evaluate the clinical and prognostic differences between LC patients with and without IPF, as well as the factors affecting prognosis in all fibrosing ILDs.

## Methods

### Study population

This retrospective single-center study included 163 consecutive patients diagnosed with ILD and LC between January 2018 and May 2023 at the Asan Medical Center in South Korea. ILD was categorized into IPF and non-IPF. IPF was diagnosed based on the diagnostic criteria of the American Thoracic Society (ATS)/European Respiratory Society (ERS)/Japanese Respiratory Society/Latin American Thoracic Association in 2018 [15]. The non-IPF type includes hypersensitivity pneumonitis, nonspecific interstitial pneumonia, smoking-related ILD, connective tissue disease-related ILD (CTD-ILD), and unclassified ILD. CTD was diagnosed by rheumatologists using specific criteria [16–21]. LC was diagnosed based on histological results, which were confirmed by pathologists at our center. Furthermore, LC was classified according to the World Health Organization tumor classification, and LC staging was performed using the 8th edition of the TNM (*T* for characteristics of the primary tumor, *N* for nodal involvement, and *M* for distant metastasis) classification of malignant tumors [22]. This study was conducted in accordance with the principles of the Declaration of Helsinki. The study protocol was approved by the Institutional Review Board of Asan Medical Center (IRB no. 2023 - 1078). Informed consent was waived due to the retrospective nature of the study and the anonymity of clinical data.

### Clinical data

The patients’ baseline characteristics, including age, sex, body mass index, smoking history, pulmonary function test results, laboratory data, and ILD and LC profiles, were obtained from their electronic medical records. Data obtained from the medical records or the National Insurance Company was used to examine mortality rates. Consistent with the recommendations of the American Thoracic Society (ATS)/European Respiratory Society (ERS), spirometry was performed to evaluate pulmonary function and measure total lung capacity and diffusing capacity for carbon monoxide (DLco) [23, 24]. The ILD profile encompassed the type, imaging and histological findings, and treatment for ILD at the time of LC diagnosis. The initial treatment for LC was categorized into four main modalities: surgery, radiotherapy, chemotherapy, and concurrent chemoradiation therapy (CCRT).

### Statistical analysis

Categorical variables were expressed as numbers and percentages, whereas continuous variables were expressed as medians with interquartile ranges. The chi-squared test or Fisher’s exact test was employed to assess the comparison of categorical variables between the two groups. Additionally, the comparison of continuous variables with normal or non-normal distribution between groups was conducted using the Student’s t-test or the Mann-Whitney U-test, respectively. The Kaplan-Meier estimate was employed for time-to-event analysis for all-cause mortality. Univariate and multivariate Cox proportional-hazards regression models were used to identify the risk factors associated with all-cause mortality. The results were expressed as a hazard ratio (HR) with a 95% confidence interval (CI). Significance was determined by two-sided *p* values < 0.05. Survival analysis was conducted using the Kaplan-Meier estimate. All statistical analyses were conducted using SPSS version 27.0 (IBM Corporation, Armonk, NY).

## Results

### Baseline patient characteristics

Among the 163 patients, 92 (56.4%) had IPF ILD and 71 (43.6%) had non-IPF ILD. The median follow-up period after LC diagnosis was 11 months. The shortest follow-up period was 1 month, and the longest duration was 64 months. The non-IPF group included patients with unclassified ILD (*n* = 54), CTD-ILD (*n* = 14), nonspecific interstitial pneumonia (*n* = 2), and chronic hypersensitivity pneumonitis (*n* = 1). The patients’ baseline characteristics at the time of LC diagnosis are summarized in Table 1. Their mean age was 70.4 years, and 92.6% and 91.4% of them were men and ever-smokers, respectively. Of the 163 patients, 141 (86.5%) were diagnosed with non-small cell lung cancer (NSCLC) and 22 (13.5%) with small cell lung cancer (SCLC). The most common histological subtype of NSCLC was adenocarcinoma (52.5%), followed by squamous cell carcinoma (45.4%) and others (2.1%). No statistically significant differences were observed in the baseline characteristics, including age, sex, and baseline pulmonary function test results, between the IPF and non-IPF groups.

**Table 1:** Baseline characteristics of patients with interstitial lung disease and lung cancer at lung cancer diagnosis

Characteristic	Total (n = 163)	IPF-LC (n = 92)	Non-IPF-LC (n = 71)	*p* Value
**Type of ILD**				
IPF	92 (56.4)	92 (100.0)	0 (0.0)	
Unclassifiable	54 (33.1)	0 (0.0)	54 (76.1)	
CTD-ILD	0 (0.0)	0 (0.0)	14 (82.4)	
NSIP	0 (0.0)	0 (0.0)	2 (11.8)	
Chronic HP	0 (0.0)	0 (0.0)	1 (5.89)	
**Age, years**	70.4 ± 7.3	70.5 ± 7.4	70.2 ± 7.2	0.827
**Male**	151 (92.6)	85 (92.4)	66 (93.0)	0.899
**BMI, kg/m** ^**2**^	24.4 ± 3.1	24.4 ± 2.8	24.4 ± 3.5	0.956
**Ever-smoker**	149 (91.4)	84 (91.3)	65 (91.5)	0.827
**Pulmonary function test**				
FVC (predicted), % (*n* = 161)	76.6 ± 16.8	76.5 ± 16.4	76.9 ± 17.5	0.884
FEV1 (predicted), % (*n* = 161)	81.2 ± 15.8	80.9 ± 15.2	81.5 ± 16.7	0.812
TLC (predicted), % (*n* = 81)	79.6 ± 13.4	79.3 ± 12.8	80.10 ± 14.47	0.800
DLco (predicted), % (*n* = 150)	55.7 ± 17.6	54.5 ± 17.7	57.39 ± 17.45	0.319
**Laboratory data**				
KL-6 ≥ 1000 U/mL (*n* = 117)	32 (27.4)	20 (28.6)	12 (25.5)	0.718
**Type of LC**				
NSCLC	141 (86.5)	81 (88.0)	60 (84.5)	0.512
Adenocarcinoma	74 (52.5)	43 (53.1)	31 (51.7)	0.867
Squamous cell carcinoma	64 (45.4)	35 (43.2)	29 (48.3)	0.546
Others^a^	3 (2.1)	3 (3.7)	0 (0.0)	0.261
SCLC	22 (13.5)	11 (12.0)	11 (15.5)	0.512

Data are expressed as mean ± standard deviation for continuous variables and number (percentage) for categorical variables. ILD, interstitial lung disease; IPF, idiopathic progressive fibrosis; CTD, connective tissue disease; HP, hypersensitivity pneumonitis; NSIP, nonspecific interstitial pneumonia; BMI, body mass index; FVC, forced vital capacity; FEV1, forced expiratory volume in 1 s; TLC, total lung capacity; DLco, diffusing capacity for carbon monoxide; KL-6, Krebs von den Lungen-6; IS, immunosuppressants, LC; lung cancer, NSCLC; non-small cell lung cancer; SCLC, small cell lung cancer

^a^ Other histological types include large cell carcinoma

### Clinical characteristics and management

Among the NSCLC patients, 32.9% were classified as stage I, 14.3% as stage II, 27.1% as stage III, and 25.7% as stage IV (Table 2). The proportions of patients who underwent surgery, chemotherapy, radiotherapy, and CCRT were 32.6%, 22.7%, 22.0%, and 5.0%, respectively. The percentage of CCRT was higher in the non-IPF group (1.2% vs. 10.0%, *p* = 0.042). In terms of ILD among the NSCLC patients, the IPF group had a higher proportion of patients who received antifibrotic agents (pirfenidone or nintedanib, 80.2% vs. 8.3%, *p* < 0.001) but had lower proportion of patients who received steroids and immunosuppressants for the initial treatment of ILD than the non-IPF group (6.2% vs. 20.0%, *p* < 0.013) (Table 2). No statistically significant difference was observed in the incidence of acute exacerbation (AE) after LC treatment (28.4% vs. 20.0%, *p* = 0.254). In addition, there was no significant difference in mortality rates between the groups (42.0% vs. 38.3%, *p* = 0.663). The clinical characteristics and treatment of SCLC patients are presented in e-Table 1. No significant differences were observed in the stage of LC, treatment for LC, and mortality between the IPF and non-IPF groups. A detailed chemotherapy regimen is summarized in e-Table 2.

**Table 2:** Comparison of the clinical characteristics and management of NSCLC patients according to the type of ILD

Characteristic	Total (n = 141)	IPF-LC (n = 81)	Non-IPF-LC (n = 60)	*p* Value
**Clinical stage of NSCLC**				0.337
I	46 (32.9)	31 (38.3)	15 (25.0)	
II	20 (14.3)	9 (11.1)	11 (18.3)	
III	38 (27.1)	21 (25.9)	17 (28.3)	
IV	36 (25.7)	10 (24.7)	17 (28.3)	
**KL-6 (U/mL),** *n*** = 98**	983.7 ± 1111.4	907.9 ± 900.1	1098.4 ± 1376.12	0.409
**Initial treatment for NSCLC**				
Surgery	46 (32.6)	30 (37.0)	16 (26.7)	0.194
Lobar resection	29 (63.0)	14 (46.7)	15 (93.8)	
Sublobar resection	17 (37.0)	16 (53.3)	1 (6.3)	
Chemotherapy	32 (22.7)	15 (18.5)	17 (28.3)	0.169
Radiotherapy	31 (22.0)	19 (23.5)	12 (20.0)	0.624
CCRT	7 (5.0)	1 (1.2)	6 (10.0)	0.042
Best supportive care	25 (17.7)	16 (19.8)	9 (15.0)	0.465
**Treatment of ILD**				
Antifibrotic agent	70 (49.6)	65 (80.2)	5 (8.3)	< 0.001
Corticosteroid ± IS	54 (38.3)	29 (35.8)^a^	25 (41.7)^b^	0.479
Initial treatment	17 (12.1)	5 (6.2)	12 (20.0)	0.013
Acute exacerbation	37 (26.2)	25 (30.9)	12 (20.0)	0.147
RT pneumonitis	5 (3.5)	2 (2.5)	3 (5.0)	0.651
**Acute exacerbation**	37 (26.2)	25 (30.9)	12 (20.0)	0.147
**Overall mortality**	57 (40.4)	34 (42.0)	23 (38.3)	0.663
AE-related death	21 (36.8)	14 (41.2)	7 (30.4)	0.409
Infection-related death	15 (26.3)	7 (20.6)	8 (34.8)	0.232
Unknown	21 (36.8)	14 (41.2)	7 (30.4)	0.409

Data are expressed as mean ± standard deviation for continuous variables and number (percentage) for categorical variables. NSCLC, non-small cell lung cancer; CCRT, concurrent chemoradiation therapy; ILD, interstitial lung disease; IS, immunosuppressants; SCLC, small cell lung cancer; AE, acute exacerbation

^a^3 patients were treated with steroid as initial treatment and during acute exacerbation

^b^2 patients were treated with steroid as initial treatment and during acute exacerbation

### Prognostic factors in NSCLC patients

Results of the Cox regression analysis of risk factors associated with mortality in NSCLC patients based on their baseline characteristics are summarized in Table 3. The results of the univariate Cox regression analysis revealed that low forced vital capacity (FVC) (HR 0.983, 95% CI 0.968-0.998, *p* = 0.023) and total lung capacity (HR 0.961, 95% CI 0.934-0.988, *p* = 0.005) were significantly correlated with mortality. Higher levels of Krebs von den Lungen-6 (KL-6) (≥ 1000) (HR 2.554, 95% CI 1.378-4.734, *p* = 0.003) and advanced clinical LC stage were associated with a higher risk of mortality. However, the subtype of ILD (IPF vs. non-IPF ILD) was not a significant factor for mortality in the univariate analysis (HR 0.892; 95% CI 0.516-1.542; *p* = 0.682). In the multivariate analysis, higher KL-6 levels were independently associated with increased mortality (HR 1.970; 95% CI 1.026-3.783; *p* = 0.042) after adjusting for other risk factors. Clinical stage was also identified as an independent risk factor for mortality (compared with stage I, HR 3.876 for stage II, *p* = 0.025, HR 5.092 for stage III, *p* = 0.002, and HR 5.626 for stage IV, *p* = 0.002).

**Table 3:** Prediction of baseline factor for mortality in patients with ILD and NSCLC assessed using the Cox proportional-hazards model

Variable	Univariate analysis			Multivariate analysis		
	HR	95% CI	*P* Value	HR	95% CI	*P* Value
Age	1.006	0.971–1.043	0.736			
Male	3.426	0.832–14.111	0.088			
Ever-smoker	1.139	0.486–2.671	0.765			
IPF (vs. non-IPF ILD)	0.963	0.563–1.649	0.892			
SqCC (vs. ADC)	1.039	0.613–1.759	0.888			
PFT						
**FVC (predicted), %**	**0.983**	**0.968–0.998**	**0.023**	0.992	0.974–1.011	0.411
FEV1 (predicted), %	0.989	0.972–1.006	0.189			
**TLC (predicted), %**	**0.961**	**0.934–0.988**	**0.005**			
DLco (predicted), %	0.986	0.973-1.000	0.051			
KL-6 ≥ 1000 U/mL	**2.554**	**1.378–4.734**	**0.003**	**1.970**	**1.026–3.783**	**0.042**
**Lung cancer stage**						
Stage I (ref)						
**Stage II**	**4.476**	**1.778–11.271**	**0.001**	**3.876**	**1.187–12.660**	**0.025**
**Stage III**	**3.722**	**1.612–8.594**	**0.002**	**5.092**	**1.801–14.401**	**0.002**
**Stage IV**	**8.717**	**3.630-20.936**	**< 0.001**	**5.626**	**1.889–16.757**	**0.002**

HR, hazard ratio; CI, confidence interval; SqCC, squamous cell carcinoma; ADC, adenocarcinoma; PFT, pulmonary function test; TLC, total lung capacity; FVC, forced vital capacity; FEV1, forced expiratory volume in 1 second; DLco, diffusing capacity for carbon monoxide; IPF, idiopathic pulmonary fibrosis; ILD, interstitial lung disease; KL-6, Krebs von den Lungen-6; IS, immunosuppressant; CCRT, concurrent chemoradiation therapy

TLC was not included in the multivariate analysis owing to its high correlation with FVC (r = 0.853, *p* < 0.001)

Bold values denote statistical significance at the *p* < 0.05 level

Table 4 presents the risk factors for all-cause mortality based on the patients’ treatment factors. In the univariate analysis, the use of steroids and/or immunosuppressants (HR 2.058, 95% CI 1.218-3.476, *p* = 0.007) and AE (HR 2.094, 95% CI 1.224-3.581, *p* = 0.007) were associated with mortality. Univariate analysis revealed that although surgery for LC was associated with lower mortality (HR 0.198, 95% CI 0.092-0.423, *p* < 0.001), chemotherapy was associated with poor prognosis (HR 2.334, 95% CI 1.276-4.269, *p* = 0.006). On the other hand, multivariate analysis revealed that only surgery was independently associated with lower mortality (HR 0.235, 95% CI 0.106-0.520, *p* < 0.001) after adjusting for other variables.

**Table 4:** Prediction of the treatment factor for mortality in patients with ILD and NSCLC assessed using the Cox proportional-hazards model

Variable	Univariate analysis			Multivariate analysis		
	HR	95% CI	*P* Value	HR	95% CI	*P* Value
ILD treatment						
Antifibrotics	0.714	0.420–1.214	0.214			
**Corticosteroid ± IS**	**2.058**	**1.218–3.476**	**0.007**	1.162	0.503–2.685	0.726
**Acute exacerbation**	**2.124**	**1.147–3.931**	**0.017**	1.282	0.542–3.034	0.572
Initial treatment for LC						
**Surgery**	**0.198**	**0.092–0.423**	**< 0.001**	**0.235**	**0.106–0.522**	**< 0.001**
**Chemotherapy**	**2.334**	**1.276–4.269**	**0.006**	1.290	0.682–2.439	0.434
Radiotherapy	0.781	0.412–1.480	0.449			
CCRT	0.916	0.222–3.776	0.904			

HR, hazard ratio; CI, confidence interval; SqCC, squamous cell carcinoma; ADC, adenocarcinoma; TLC, total lung capacity; FVC, forced vital capacity; FEV1, forced expiratory volume in 1 second; DLco, diffusing capacity for carbon monoxide; IPF, idiopathic pulmonary fibrosis; ILD, interstitial lung disease; KL-6, Krebs von den Lungen-6; IS, immunosuppressant; CCRT, concurrent chemoradiation therapy

Bold values denote statistical significance at the *p* < 0.05 level

### Comparison of clinical characteristics and clinical course according to baseline KL-6 levels

Because KL-6 was independently associated with mortality in NSCLC patients, Table 5 presents a comparison of clinical characteristics according to KL-6 levels. No significant differences were observed in the proportion of ILD subtypes, sex, body mass index, and smoking history between the two groups. Patients with higher KL-6 levels (67.5 vs. 71.1 years, *p* = 0.030) had lower mean age, predicted FVC values (72.9% vs. 83.4%, *p* = 0.004), and DLco (46.1% vs. 58.7%, *p* = 0.001) than those with lower KL-6 levels. No differences were observed between the groups in terms of histological type and LC stage. Although there were no statistically significant differences in stage between the two groups, there seemed to be a tendency, especially in stage I, for the low KL-6 (< 1000 U/mL) group to have a higher proportion of surgical cases. In terms of treatment for ILD and LC, the use of steroid and/or immunosuppressants was more common in patients with higher KL-6 levels (69.0% vs. 27.5%, *p* < 0.001) than in those with lower levels. The incidence of AE (51.7% vs. 20.3%, *p* = 0.002) and mortality risk (65.5% vs. 31.9%, *p* = 0.002) were higher in patients with higher KL-6 levels than in those with lower levels.

**Table 5:** Comparison of clinical characteristics in NSCLC patients with fibrosing ILD according to KL-6

Characteristic	KL-6 ≥ 1,000 (n = 29)	KL-6 < 1,000 (n = 69)	*P* Value
**Type of ILD**			0.807
IPF	18 (62.1)	41 (59.4)	
Non-IPF ILD	11 (37.9)	28 (40.6)	
**Age, years**	67.5 ± 7.1	71.1 ± 6.9	0.030
**Male**	27 (93.1)	64 (92.8)	0.951
**BMI, kg/m** ^**2**^	24.0 ± 3.58	24.7 ± 3.2	0.899
**Ever-smoker**	27 (93.1)	64 (92.8)	0.516
**Pulmonary function test**			
FVC (predicted). % (*n* = 97)	68.3 ± 17.3	79.0 ± 16.0	0.007
FEV1 (predicted), % (*n* = 97)	72.9 ± 15.6	83.4 ± 15.5	0.004
TLC (predicted), % (*n* = 56)	72.5 ± 13.9	83.6 ± 11.2	0.003
DLco (predicted), % (*n* = 56)	46.1 ± 16.5	58.7 ± 16.0	0.001
**Type of NSCLC**			0.668
Adenocarcinoma	17 (58.6)	34 (49.3)	
Squamous cell carcinoma	11 (37.9)	34 (49.3)	
Others	1 (3.5)	1 (1.4)	
**Stage of NSCLC**			0.120
I	5 (17.2)	28 (40.6)	
II	7 (24.1)	9 (13.0)	
III	7 (24.1)	16 (23.2)	
IV	10 (34.5)	16 (23.2)	
**Initial treatment for NSCLC**			
Surgery	6 (20.7)	26 (37.7)	0.102
Chemotherapy	9 (31.0)	14 (20.3)	0.252
Radiotherapy	5 (17.2)	17 (24.6)	0.423
CCRT	1 (3.4)	3 (4.3)	0.837
Best supportive care	8 (27.6)	9 (13.0)	0.083
**ILD treatment**			
Antifibrotics	16 (55.2)	40 (58.0)	0.798
Corticosteroid ± IS	20 (69.0)^a^	25 (36.2)^b^	0.003
Initial treatment	8 (27.6)	7 (10.1)	0.029
Acute exacerbation	15 (51.7)	16 (23.2)	0.006
RT pneumonitis	0 (0.0)	3 (4.3)	0.254
**Acute exacerbation**	15 (51.7)	14 (20.3)	0.002
**Overall mortality**	19 (65.5)	22 (31.9)	0.002
AE-related death	11 (57.9)	6 (27.3)	0.047
Infection-related death	2 (10.5)	6 (27.3)	0.249
Unknown	6 (31.6)	10 (45.5)	0.364

Data are expressed as mean ± standard deviation for continuous variables and number (percentage) for categorical variables. KL-6, Krebs von den Lungen-6; ILD, interstitial lung disease; IS, immunosuppressants; BMI, body mass index; FVC, forced vital capacity; FEV1, forced expiratory volume in 1 s; TLC, total lung capacity; DLco, diffusing capacity for carbon monoxide; NSCLC, non-small cell lung cancer; LC, lung cancer; CCRT, concurrent chemoradiation therapy

^a^3 patients were treated with steroid as initial treatment and during acute exacerbation

^b^1 patient were treated with steroid as initial treatment and during acute exacerbation

### Survival analysis

**Fig. 1:**
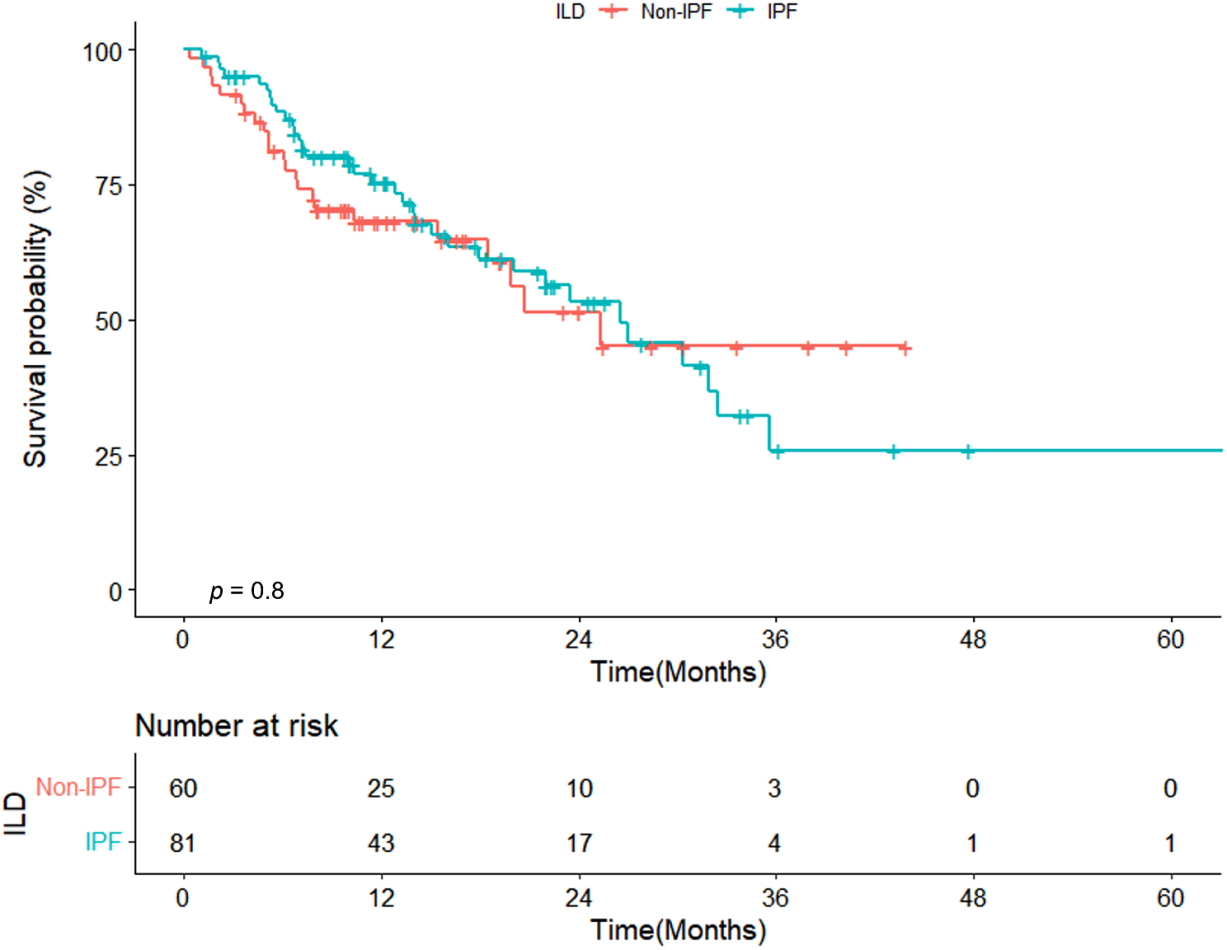
Comparison of survival curves between IPF and non-IPF patients with NSCLC

**Fig. 2:**
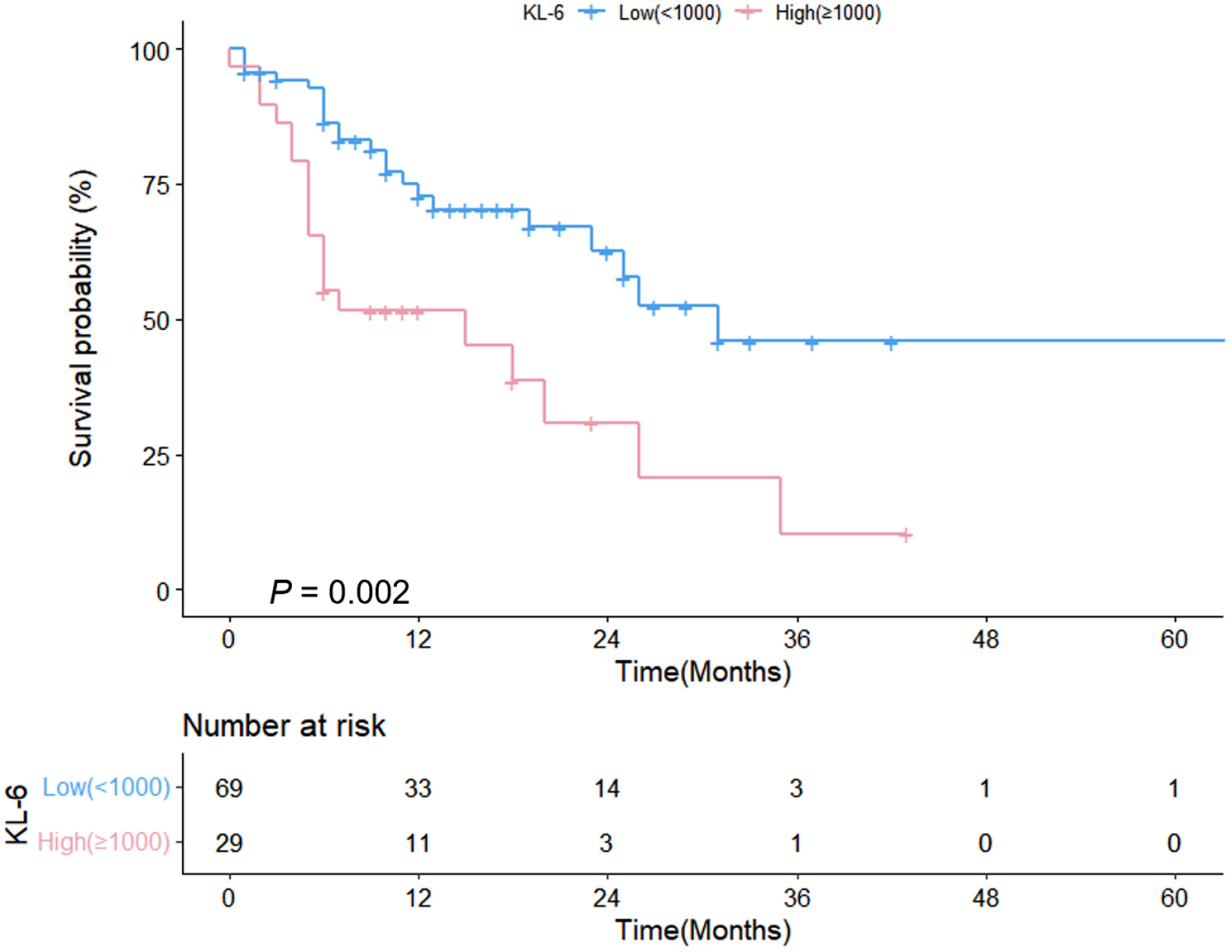
Comparison of survival curves between patients with higher (≥ 1000U/mL) and lower (< 1000U/mL) KL-6 levels among NSCLC patients

Figure 1 presents a comparison of survival curves between IPF and non-IPF patients with NSCLC. No statistically significant difference was observed in mortality between the two groups (median survival: 26 vs. 25 months, *p* = 0.08, Fig. 1). Figure 2 illustrates a comparison of survival curves based on the KL-6 level. Median survival was shorter in patients with higher KL-6 levels (≥ 1000) (15 vs. 31 months, respectively, *p* = 0.002) than in those with lower levels (< 1000). e-Fig. 1 shows a comparison of survival curves for patients with SCLC, distinguishing between IPF and non-IPF patients.

## Discussion

In the present study, no significant differences were observed in the frequency of AE and prognosis between IPF and non-IPF ILD in patients with LC. Higher KL-6 levels (≥ 1000) were independently associated with mortality in LC patients with fibrosing ILD, along with the LC stage. Furthermore, patients with higher KL-6 levels had lower survival rates.

Generally, IPF patients exhibits a poorer prognosis than non-IPF ILD patients [25], however in this study, no difference was observed in the prognosis between IPF and non-IPF ILD patients with LC. There were some previous studies that focused on the prognosis according to the ILD subtype in ILD patients with LC. Yoon et al. [26] have previously reported that LC with IPF had higher mortality than LC in non-IPF ILD (HR 6.2, *p* = 0.001) among 31 IPF-LC patients and 16 non-IPF ILD–LC patients. However, a meaningful comparison between the groups was difficult due to the small sample size and difference in cancer subtypes; despite being statistically insignificant, the proportions of patients with squamous cell carcinoma and adenocarcinoma were 41% and 26% in the IPF group and 19% and 63% in the non-IPF ILD group, respectively. Furthermore, other studies have reported that non-IPF ILD patients with LC have a poorer prognosis than those without [27]. These results suggest that even in non-IPF ILD, the development of LC may lead to a poor prognosis, which is consistent with the results of our study. In addition to IPF, ILDs have other subtypes, such as progressive fibrosing interstitial lung disease and progressive pulmonary fibrosis, which exhibit a progressive course [28, 29]. As it is possible that some patients with progressive pulmonary fibrosis were included in the non-IPF ILD group, this could be an additional factor contributing to the absence of prognostic differences between the IPF and non-IPF groups. It is also possible that the use of antifibrotic agents, which were more frequently used in IPF [30], may not lead to any differences in prognosis between the two groups. Antifibrotic agents have been demonstrated to effectively decelerate the progression of fibrosis and the effect, though limited, of antitumor properties [31, 32]. The effects of medication may have contributed to the improvement in the survival rates of the IPF group. Another possible explanation for the absence of prognostic differences between IPF and non-IPF ILD with LC is the incidence of AE in both IPF and non-IPF ILD groups [33, 34], which have been associated with poor clinical outcomes. In our study, the incidence rates of AE in IPF and non-IPF patients were 30.9% and 20.0%, respectively (*p* = 0.147), among those with NSCLC.

In the present study, an independent association between KL-6 level and poor prognosis was observed in LC patients with fibrosing ILD. Injury, cell proliferation, and inflammation lead to the disruption of alveolar epithelial cells and the diffusion of KL-6 into the pulmonary epithelial lining fluid and blood [35]. KL-6 has been suggested to be a diagnostic and prognostic indicator not only in IPF but also in non-IPF ILD [36, 37]. Previous studies have reported that baseline serum KL-6 levels might act as a sensitive predictor of AE onset in IPF [38]. Elevated KL-6 levels have also been reported to be associated with more severe, more progressive, and poorer outcomes of ILD [39]. In addition, previous studies demonstrated that high KL-6 levels were associated with poor clinical outcomes in NSCLC patients who underwent surgery or received tyrosine kinase inhibitor treatment [40, 41]. Recently, there have also been studies that have presented KL-6 as a prognostic factor in LC patients treated with immune checkpoint inhibitors [42]. Based on these previous reports, KL-6 might serve as a significant biomarker in LC patients with ILD. A study by Tomita et al. [43] that included 14 ILD patients with NSCLC reported that high KL-6 levels exhibited a trend indicating a worse prognosis compared with low KL-6 levels (*p* = 0.063). However, a study by Miyazaki et al. [44] on 273 LC patients with and without ILD reported that KL-6 levels were higher in the ILD group, but no significant difference was observed in prognosis based on KL-6 levels; this could be due to the low cutoff value (500 U/mL) and small sample size (*n* = 68). However, in our study, the group with higher KL-6 levels (≥ 1000 U/mL) showed higher mortality in LC patients with fibrosing ILD. In the lower KL-6 group, there was a relatively higher proportion of patients undergoing surgery, and this might have influenced the prognosis. Focusing on patients with KL-6 values, Kaplan-Meier curves were plotted for stages I and II in surgically treated patients and for stages III and IV in patients receiving chemotherapy. Among patients undergoing surgery in stages I and II, those with higher KL-6 levels exhibited higher mortality rates compared to those with lower KL-6 levels (*p* = 0.044, e-Fig. 2). In patients receiving chemotherapy in stages III and IV, although no statistically significant difference was observed, there was a trend of worse prognosis in the higher KL-6 group compared to the lower KL-6 group (*p* = 0.07, e-Fig. 3). While the sample size was not sufficient for subgroup analysis or propensity score matching, there seemed to be a discernible difference in prognosis based on KL-6 values in patients with similar stages and treatments. Therefore, KL-6 could act as a prognostic factor. These findings suggest that KL-6 with an appropriate cutoff level is a potent prognostic biomarker in these patients.

In this study, the LC stage and surgery performed for LC were the independent prognostic factors in NSCLC patients. Even in LC patients with ILD, it is relatively well known that the clinical stage of LC is one of the prognostic factors in these patients. Sato et al. reported that the 5-year survival rates after surgical resection in ILD patients with LC were 59%, 42%, 43%, 29%, 25%, 17%, and 16% for stages (TNM stage, 6th edition) IA, IB, IIA, IIB, IIIA, IIIB, and IV, respectively [45]. A study by Alomaish et al. [46] on 146 ILD patients with LC reported that patients with stage IA, IB, IIB, and IIIA LC had a significantly lower risk of mortality or higher survival than those with stage IV LC based on the 7th edition of the TNM system (HR 0.121, 0.270, 0.273, and 0.362, respectively). In our study, the clinical stage of LC was also an independent prognostic factor. Furthermore, surgery was independently associated with a favorable outcome in our study. Likewise, in a study by Han et al. [47] on 160 patients diagnosed with LC and IPF, the patients were divided into the Gender-Age-Physiology stage and LC clinical stage; it was found that in Gender-Age-Physiology stage I, surgery significantly improved survival in patients with early and advanced LC stages (*p* = 0.023 and *p* = 0.019, respectively). In one survey, 78.2% of physicians responded that they consider surgery in a patient with IPF of mild-to-moderate functional impairment (FVC > 50%, DLco > 35%) with operable NSCLC (TNM stages I-II) [48]. Although there is a risk of AE in about 10% of patients who receive surgery for LC [49], and surgery is feasible in selective patients with an early stage of LC and relatively preserved pulmonary function [50]. 

This study has some limitations. First, it was a single-center, retrospective study, which might have resulted in selection bias. Some of our patients diagnosed with unclassifiable IIP underwent multidisciplinary discussion, but accurate classification was challenging. If there had been a larger number of patients, meaningful comparisons could have been possible for each ILD subtype. However, our main goal in this study was to observe the differences between IPF and non-IPF ILD in patients with coexisting lung cancer. Second, it focused on only ILD patients diagnosed with LC, and there may be limitations in the presentation of cancer prevalence among ILD subtypes. Third, the follow-up periods were relatively short, with the median follow-up period being 11 months after cancer diagnosis. However, considering the poor prognosis of patients with ILD-LC, it is believed that a meaningful analysis is warranted. Although there were no well-conducted large-scale studies, the prognosis for ILD-LC is poor, with a median survival of only 15 months, as referenced in *PLos One. 2021; 16(9): e0255375* (Reference 46). Although there was a limitation in that the follow-up period was short, considering the poor prognosis, it was thought that the difference in prognosis between IPF-LC and non-IPF-LC could be analyzed in this study. Indeed, despite the short follow-up observation in our study, a total of 67 (41.1%) out of 163 patients have died, with 45 (27.6%) of them having died before the median follow-up of 11 months. One of the weaknesses of our study was the small number of patients. One of the weaknesses of our study was the small number of patients. If there had been a larger number of patients, it would have been possible to conduct additional analysis, adjusting for stage, treatment, and confounding variables. Finally, to analyze the accurate impact of KL-6 on prognosis, a method such as propensity matching that adjusts for stage and treatment history would have been necessary, but such analysis was not possible due to the small number of patients. Future research with large-scale patients will be needed in the future to confirm these results. Despite these limitations, we believe that the strength of our study is the presentation of LC characteristics and clinical course based on ILD subtypes.

## Conclusions

In conclusion, no statistically significant differences were observed in clinical characteristic s and mortality between IPF and non-IPF ILD patients with LC. This finding suggests that diagnosis and management of LC are important in both patient groups. Furthermore, KL-6 might serve as a prognostic biomarker in LC patients with fibrosing ILD.

### Electronic supplementary material

Below is the link to the electronic supplementary material.

Supplementary Material 1

## Data Availability

The datasets utilized and/or examined in the present study can be obtained from the corresponding author upon a reasonable request.

## Abbreviations

ADC: adenocarcinoma
AE: acute exacerbation
AEC: alveolar epithelial cell
ATS: American Thoracic Society
BMI: body mass index
CI: confidential interval
CTD: connective tissue disease
DLco: diffusing capacity for carbon monoxide
ERS: European Respiratory Society
GAP: Gender–Age–Physiology
FVC: forced vital capacity
HP: hypersensitivity pneumonitis
HR: hazard ratio
ICI: immune checkpoint inhibitor
ILD: interstitial lung disease
IPF: idiopathic pulmonary fibrosis
KL-6: Krebs von den Lungen-6
LC: lung cancer
NSCLC: non-small cell lung cancer
NSIP: nonspecific interstitial pneumonia
PF-ILD: progressive fibrosing interstitial lung disease
SCLC: small cell lung cancer
SqCC: squamous cell carcinoma
TLC: total lung capacity
TNM: *T* for characteristics of the primary tumor, *N* for nodal involvement, and *M* for distant metastasis
