# Supplementary material for: Clinical characteristics and outcome of lung cancer in patients with fibrosing interstitial lung disease
Source: BMC Pulm Med. 2024 Mar 15;24:136. doi: 10.1186/s12890-024-02946-6 (PMC10943814; doi:10.1186/s12890-024-02946-6)
Supplement: Supplementary file 1 — Supplementary Material 1 [file 12890_2024_2946_MOESM1_ESM.docx]

**Clinical characteristics and outcome of lung cancer in patients with fibrosing interstitial lung disease**

**Author list**
Soo Jin Han^1^, Hyeon Hwa Kim^1^, Dong-gon Hyun^1^, Wonjun Ji^1^, Chang-Min Choi^1,2^, Jae Cheol Lee^2^, Ho Cheol Kim^1^

^1^ Department of Pulmonary and Critical Care Medicine, Asan Medical Center, University of Ulsan College of Medicine, Seoul, Republic of Korea

^2^ Department of Oncology, Asan Medical Center, University of Ulsan College of Medicine, Seoul, Republic of Korea

**Supplemental Material**

**e-Table 1.** Comparison of lung cancer characteristics in ILD patients with SCLC according to the type of ILD.

| Characteristic | All  (n = 22) | IPF-LC  (n = 11) | Non-IPF-LC  (n = 11) | *P* Value |
| --- | --- | --- | --- | --- |
| **Clinical stage** |  |  |  | >0.999 |
| Limited | 12 (54.5) | 6 (54.5) | 6 (54.5) |  |
| Extensive | 10 (45.5) | 5 (45.5) | 5 (45.5) |  |
| **Initial treatment for lung cancer** |  |  |  |  |
| Surgery | 1 (4.5) | 1 (9.1) | 0 (0.0) | >0.999 |
| Chemotherapy | 13 (59.1) | 7 (63.6) | 6 (54.5) | >0.999 |
| Radiotherapy | 2 (9.1) | 1 (9.1) | 1 (9.1) | >0.999 |
| Concurrent chemoradiation therapy | 4 (18.2) | 1 (9.1) | 3 (27.3) | 0.586 |
| Best supportive care | 2 (9.1) | 1 (9.1) | 1 (9.1) | >0.999 |
| **Treatment of ILD** |  |  |  |  |
| Antifibrotic agent | 11 (50.0) | 10 (90.9) | 1 (9.1) | <0.001 |
| Corticosteroid ± IS | 10 (45.5) | 5 (45.5) | 5 (45.5) | >0.999 |
| Initial treatment | 4 (18.2) | 0 (0.0) | 4 (36.4) | 0.090 |
| Acute exacerbation | 9 (40.9) | 5 (45.5) | 4 (36.4) | >0.999 |
| **Acute exacerbation** | 9 (40.9) | 5 (45.5) | 4 (36.4) | >0.999 |
| **Overall mortality** | 11 (50.0) | 4 (36.4) | 7 (63.6) | 0.201 |
| AE-related death | 2 (18.2) | 2 (50.0) | 0 (0.0) | 0.109 |
| Infection-related death | 2 (18.2) | 0 (0.0) | 2 (18.2) | 0.491 |
| Unknown | 7 (63.6) | 2 (50.0) | 5 (71.4) | 0.576 |

Data are expressed as mean ± standard deviation for continuous variables and number (percentage) for categorical variables. ILD, interstitial lung disease; SCLC, small cell lung cancer; IS, immunosuppressants; AE, acute exacerbation

**e-Table 2-1.** The regimen of chemotherapies in patients with IPF-LC according to the type of lung cancer

|  | **IPF-LC** | | | |
| --- | --- | --- | --- | --- |
| **Regimen** | **All**  **(n = 22)** | **ADC**  **(n = 9)** | **SqCC**  **(n = 6)** | **SCLC**  **(n = 7)** |
| Pemetrexed | 0 (0.0%) | 0 (0.0%) | 0 (0.0%) | 0 (0.0%) |
| Pembrolizumab | 0 (0.0%) | 0 (0.0%) | 0 (0.0%) | 0 (0.0%) |
| Pemetrexed & Carboplatin or Cisplatin | 3 (13.6%) | 3 (33.3%) | 0 (0.0%) | 0 (0.0%) |
| Permbrolizumab & Pemetrexed | 0 (0.0%) | 0 (0.0%) | 0 (0.0%) | 0 (0.0%) |
| Gemcitabine & Cisplatin | 1 (4.5%) | 0 (0.0%) | 1 (16.7%) | 0 (0.0%) |
| Vinorelbine & Cisplatin | 0 (0.0%) | 0 (0.0%) | 0 (0.0%) | 0 (0.0%) |
| Etoposide & Carboplatin or Cisplatin | 4 (18.2%) | 0 (0.0%) | 1 (16.7%) | 3 (42.9%) |
| Paclitaxel & Carboplatin | 0 (0.0%) | 0 (0.0%) | 0 (0.0%) | 0 (0.0%) |
| Atezolizumab & Etoposide & Carboplatin | 0 (0.0%) | 0 (0.0%) | 0 (0.0%) | 0 (0.0%) |
| Pembrolizumab & Paclitaxel & Carboplatin | 8 (36.4%) | 0 (0.0%) | 4 (66.6%) | 4 (57.1%) |
| Pembrolizumab & Pemetrexed & Carboplatin | 6 (27.3%) | 6 (66.7%) | 0 (0.0%) | 0 (0.0%) |

ADC, adenocarcinoma; Sqcc, Squamous cell carcinoma; SCLC, small cell lung cancer.

**e-Table 2-2** The regimen of chemotherapies in patients with Non IPF-LC according to the type of lung cancer

|  | **Non IPF-LC** | | | |
| --- | --- | --- | --- | --- |
| **Regimen** | **All**  **(n = 23)** | **ADC**  **(n = 9)** | **SqCC**  **(n = 8)** | **SCLC**  **(n = 6)** |
| Pemetrexed | 1 (4.4%) | 1 (11.1%) | 0 (0.0%) | 0 (0.0%) |
| Pembrolizumab | 5 (21.7%) | 2 (22.2%) | 3 (37.5%) | 0 (0.0%) |
| Pemetrexed & Carboplatin or Cisplatin | 0 (0.0%) | 0 (0.0%) | 0 (0.0%) | 0 (0.0%) |
| Permbrolizumab & Pemetrexed | 2 (8.7%) | 2 (22.2%) | 0 (0.0%) | 0 (0.0%) |
| Gemcitabine & Cisplatin | 0 (0.0%) | 0 (0.0%) | 0 (0.0%) | 0 (0.0%) |
| Vinorelbine & Cisplatin | 0 (0.0%) | 0 (0.0%) | 0 (0.0%) | 0 (0.0%) |
| Etoposide & Carboplatin or Cisplatin | 4 (17.4%) | 1 (11.1%) | 0 (0.0%) | 3 (50.0%) |
| Paclitaxel & Carboplatin | 0 (0.0%) | 0 (0.0%) | 0 (0.0%) | 0 (0.0%) |
| Atezolizumab & Etoposide & Carboplatin | 3 (13.0%) | 0 (0.0%) | 0 (0.0%) | 3 (50.%) |
| Pembrolizumab & Paclitaxel & Carboplatin | 5 (21.7%) | 0 (0.0%) | 5 (62.5%) | 0 (0.0%) |
| Pembrolizumab & Pemetrexed & Carboplatin | 3 (13.0%) | 3 (33.3%) | 0 (0.0%) | 0 (0.0%) |

ADC, adenocarcinoma; Sqcc, Squamous cell carcinoma; SCLC, small cell lung cancer.


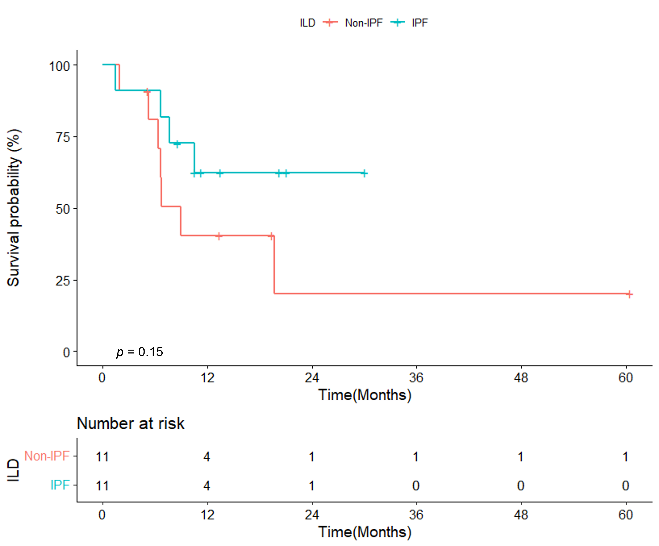


e-Figure 1. Comparison of survival curves between IPF and non-IPF patients with SCLC. The red line represents non-IPF patients, while the blue line represents IPF patients. The survival curve is generated using the Kaplan-Meier methods, and the *p*-value corresponds to the log-rank test.


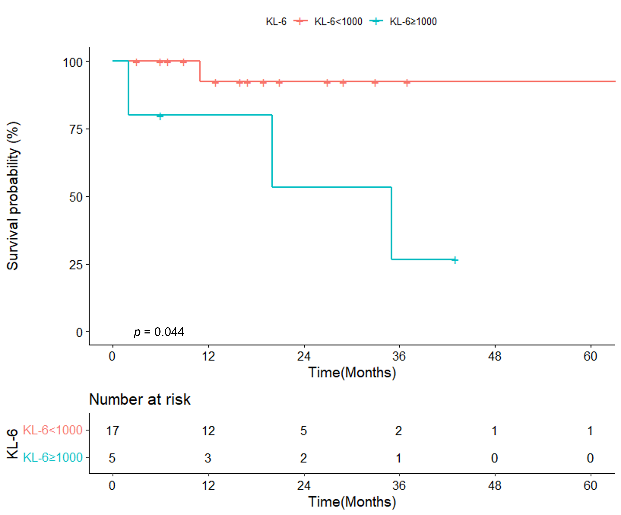


e-Figure 2. Comparison of survival curves for patients with stage I and II NSCLC who underwent surgery, categorized based on KL-6 levels. The red line represents the low KL-6 group (<1000), while the blue line represents the high KL-6 group (≥1000). The *p*-value corresponds to the log-rank test.


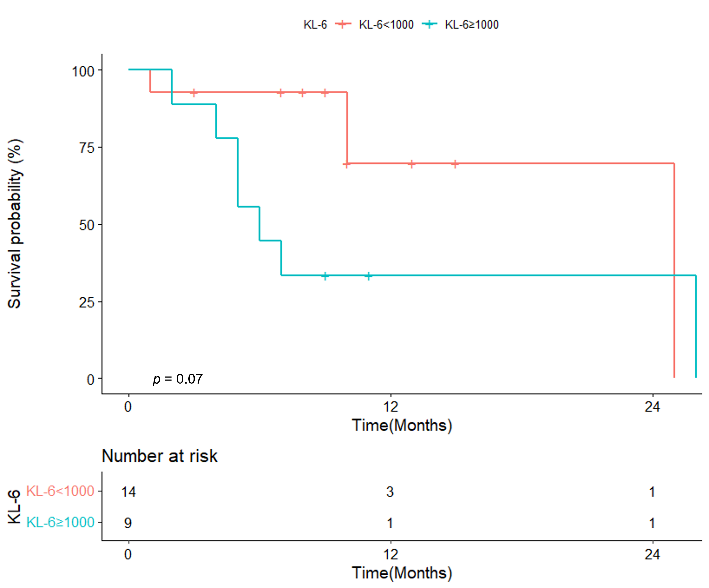


e-Figure 3. Comparison of survival curves for patients with stage III and IV NSCLC who received chemotherapy, based on KL-6 levels. The red line represents the low KL-6 group (<1000), while the blue line represents the high KL-6 group (≥1000). The *p*-value corresponds to the log-rank test.
